# Contributions to the Knowledge of the Microscopic Characters of the Mucous Membrane of the Respiratory Organs and Its Products

**Published:** 1844-04

**Authors:** 


### Art. XVIII.

*Beiträge zur Kenntniss der Kranken Schleimhaut der Respirationsorgane und ihrer Producte durch das Mikroskop.* Von Fr. Bühlmann.—*Bern*, 1843. Mit Taf. 3.

*Contributions to the Knowledge of the Microscopic Characters of the Mucous Membrane of the Respiratory Organs and its Products.* By Fr. Bühlmann.—*Bern*, 1843. 4to, pp. 82. With Three Plates.

There is not much that is new or very important in this work, (the author's inaugural dissertation for the doctor's degree at Bern;) but all "contributions" to the knowledge of the diseases of the respiratory organs, already so carefully studied without the microscope, must be thankfully received.

The first section of the first part of the dissertation contains a description of the external characters of sputa; the second is given to the normal products of mucous membranes. Neither of these has anything important in it. In the third is an examination of the morbid products of the respiratory mucous membrane which the author describes under twelve heads, as follows:

1. *Pus and its corpuscles*, which he considers (as his friends Valentin and Gerber do) to be modified exudation- (lymph-) corpuscles, having, as intermediate stages in development or degradation, the various forms of mucous corpuscles. His description of the corpuscles themselves has nothing novel; but of the diseases in which he finds pus, he gives a longer list than is usually admitted.

"In consequence of irritation, exudation-corpuscles form, which, by division and by the taking-up of fatty matter, pass through the middle stage of the so-called mucous corpuscles into the proper pus-corpuscles. These middle states we find especially in slight degree of coryza, after the first excitement is over; at the commencement of all catarrhs; in slight forms of bronchitis on the second or third day after secretion has begun. Completely formed pus-corpuscles are found in all chronic catarrhs in a later stage when the secretion, at first transparent, begins to grow turbid and yellowish; and in the second stage of every severe nasal catarrh; in these they form with especial rapidity..... We find pus, moreover, in the second stage of every severe bronchial catarrh or bronchitis; and in phthisis, in the stage of crude tubercles as well as with open cavities. .... In chronic pneumonia also, and in bronchorrhea, there are pus-corpuscles in great number. There are few chronic catarrhs in which the expectoration does not consist of completely purulent constituents mixed with the middle states of pus, the mucus-corpuscles. As soon as grayish or yellowish spots form in the frothy transparent catarrhal expectoration, these consist, for the most part, of pus. The yellowish, copious, homogeneous sputa of the consistence of a balsam, which one sees in chronic catarrh, and which are exactly like the expectoration of phthisical patients, always contain the most perfect pus." (pp. 38-9.)

2. *Morbid epithelia.* The author says that in irritation of the respiratory mucous membrane there is never a profuse shedding of the epithelium previous to the discharge of exudation- or mucus-corpuscles; and he fairly advances this as strong evidence against the notion of Vogel and Henle, that these corpuscles, as well as the pus-corpuscles, are formed from the younger epithelial cells; because these could not appear till the old ones had been cast off.

All kinds of epithelia, and in all their stages of development, are found in sputa; and sometimes their forms appear altered by disease; but the several changes of form cannot be connected with any corresponding forms of disease. The author says, however,

"I have always found a peculiar kind of ciliary epithelium at the beginning of common nasal catarrh. .... Its form is truly protean as long as the ciliary movement continues. The cells are most frequently roundish, often quite circular, often conical with either lateral or central ciliæ; sometimes they are long and divided in several parts; the same cell which in one moment is exactly circular, with a central nucleus and tuft-like ciliæ, is in the next turned round, and now is a ciliary cylinder or an oval or conical body. The ciliæ are like those fully developed, but longer; and they turn in the most beautiful flapping manner from right to left, and in the reverse direction, making each time two distinct bends... .. The roots of the ciliæ are here, also, very distinct; they are thicker than the ciliæ themselves, and contain nuclei, but no nucleoli. The colour of the cells is pale and clear; and they are transparent as far as the semi-transparent part on which the roots of the ciliæ are seated..... The existence of cells of this kind is short and very transitory; they are hardly found before they gradually diminish in number, and they disappear completely as soon as the first violence of the coryza has passed off; which often takes place in twenty-four hours, and sometimes even in twelve hours." (pp. 41-2.)

Their nature and their origin and mode of formation are at present obscure.

3. *Albumen-granules.* These, and the large granular corpuscles, (Gluge's *compound inflammatory globules*,) formed by aggregation of granules, are very rarely found in the acute diseases of the respiratory mucous membrane, but are abundant in chronic bronchitis, phthisis, bronchial blennorrhea, and generally in the expectoration of persons whose blood is defective in fibrin, and in that of chlorotic persons.

4. *Exudation-corpuscles*, such as first received this name from Valentin, and such as Gerber supposes to be the normal organized deposit from living fibrinous fluids, are found abundantly in the products of disease of the mucous membrane of the respiratory organs, nor can there be any better occasion for observing them than is afforded in the early stages of a common coryza. They are at first spherical, but at a later period, lens- or cake-shaped nuclei, yellowish-white or pale red, and containing nucleoli. In size they vary from 1-400 to 1-320th of a line in diameter. By mutual pressure and adaptation they become polygonal. In this state they may be stationary; but when more highly developed they become cells; and when retrograding they pass (apparently very quickly) into the state of pus-corpuscles. In the transition to these states their forms are variously altered; and, especially, though at first their surface is smooth and uninterrupted, they become as if grooved and cracked. The diseases in which these corpuscles are most abundantly found are nasal catarrh, the more acute bronchial catarrh (even from its commencement), and the early stages of phthisis.

5. *Blood-corpuscles*, in their normal or in variously altered forms, are found much more commonly than they can be discerned by the unassisted eye. "In pneumonia, when the blood at first is not yet intimately mingled with the rest of the sputa, the blood-corpuscles are quite normal, or at most somewhat swollen; but when the mixture is fully effected they are almost always much altered, and in the *prune-juice* expectoration, nothing can be found of them but rudiments of nuclei, and the fluid coloured by them." (p. 53.)

6. *Melanotic structures.* These occur in two forms: First, as cells containing points of black pigment; and secondly, as loose pigment. The cells are very like small tesselated epithelium-cells; some not larger than blood-corpuscles, others as large as the epithelium-cells of the mouth. The pigment, which appears to be formed in the cells as they are being developed, is distributed in them not uniformly, but in patches. In the most developed cells, the dark particles produce complete blackness; and some of them are elongated into the form of caudate bodies. Loose black pigment granules are also often found in sputa, of the same kind as those in the cells. Both together occur very frequently in phthisical expectoration, especially in patients who have cavities, in whom they are probably derived from the ulcerated pulmonary tissue. Pigment of the same kind is also found abundantly in diseased black bronchial glands.

7. *Tuberculous matter.* On this the author gives the descriptions of his predecessors, and acknowledges that he cannot clear up the obscurity which rests upon the subject, concluding only that the *tuberculositas pulmonum* cannot be discerned by any peculiar microscopic tuberculous substance, and that there is no substance found in the expectoration of phthisical patients which does not also sometimes occur in those otherwise affected.

8. *Crystals* of the various salts contained in the secretions and crystals of cholestearine are often found abundantly in expectoration; but they appear to be subject to no other general rule than that they are usually most numerous in sputa which have lain long, and been permitted to evaporate their fluid parts in the air-passages.

9. *Fatty matters,* in globules of oil, and in particles of stearine are also often found in phthisical and other kinds of expectoration. The consistent cerate-like fatty matters containing most stearine appear to be products of disease of the mucous glands, and are especially abundant in chronic bronchitis.

10, 11, 12. Under these heads the author mentions the occurrence in the expectorated fluids of portions of the normal tissues destroyed by sloughing or ulceration, portions of morbid growths from the mucous membrane, and various accidentally mixed substances; but we need not extract any of this portion of his treatise.

In the second part of the work he speaks of the microscopic examination of the mucous membrane of the respiratory organs in the diseased state; and in the third part he gives briefly the results of his observations. These, as he himself admits, are few and unsatisfactory, little better than some probable negatives, which our readers may easily themselves draw from the analysis which we have given. We are bound to add, however, that the merit of the author is not to be measured by the importance of his results; he must have worked hard to produce such a dissertation; and his honesty must be praised since it has prevented his attempting to make that clear by unfair means which is in itself obscure and uncertain.

